# The Impact of Spatial Frequency on the Perception of Crowd Emotion: An fMRI Study

**DOI:** 10.3390/brainsci13121699

**Published:** 2023-12-09

**Authors:** Dongfang Zhao, Xiangnan Shen, Shuaixia Li, Weiqi He

**Affiliations:** 1 Research Center of Brain and Cognitive Neuroscience, Liaoning Normal University, Dalian 116029, China; eiffel_611021@sina.com (D.Z.); shenxiangnan@hcmedx.cn (X.S.); lsx91@lnnu.edu.cn (S.L.); 2 Key Laboratory of Brain and Cognitive Neuroscience, Liaoning Province, Dalian 116029, China

**Keywords:** ensemble coding, spatial frequency, fMRI, crowd faces, crowd emotion

## Abstract

Recognizing the emotions of faces in a crowd is crucial for understanding overall behavior and intention as well as for smooth and friendly social interactions. However, it is unclear whether the spatial frequency of faces affects the discrimination of crowd emotion. Although high- and low-spatial-frequency information for individual faces is processed by distinct neural channels, there is a lack of evidence on how this applies to crowd faces. Here, we used functional magnetic resonance imaging (fMRI) to investigate neural representations of crowd faces at different spatial frequencies. Thirty-three participants were asked to compare whether a test face was happy or more fearful than a crowd face that varied in high, low, and broad spatial frequencies. Our findings revealed that fearful faces with low spatial frequencies were easier to recognize in terms of accuracy (78.9%) and response time (927 ms). Brain regions, such as the fusiform gyrus, located in the ventral visual stream, were preferentially activated in high spatial frequency crowds, which, however, were the most difficult to recognize behaviorally (68.9%). Finally, the right inferior frontal gyrus was found to be better activated in the broad spatial frequency crowds. Our study suggests that people are more sensitive to fearful crowd faces with low spatial frequency and that high spatial frequency does not promote crowd face recognition.

## 1. Introduction

Imagine walking down the street and suddenly seeing a crowd of fearful faces signaling threats, prompting quick evacuation. During this period, emotional information from people’s faces is rapidly summarized and understood by the brain. The emotion of a crowd spreads fast, and the mood of one member tends to be transmitted to others [1,2]. Crowd emotions not only regulate group-level behavior and promote social harmony but also guide, motivate, and predict individual behavior [3,4]. For example, sad [5], fearful [6], and proud crowds [7] can intensify or weaken people’s responsibility consciousness and realign both their expressions and behavior in accordance with the overall standards, thus ensuring smooth intergroup interaction.

Haberman and Whitney [8] observed that humans can extract mean emotions from multiple faces using ensemble coding, in which the visual information of multiple similar objects is averaged and integrated to form a general representation of the crowd [9,10,11]. This mechanism enhances visual cognition for coping with a variety of features, forming a compressed and efficient representation of information [9]. Similar to the “threat superiority effect”, threatening faces (e.g., fearful or angry faces) are processed more efficiently than the positive or neutral ones in individual facial expression [12,13,14]. Research found that participants were more likely to classify ambiguous crowd faces as angry compared to happy faces [15]. However, Bucher and Voss [16] showed that happy crowds have a higher accuracy and longer gaze time, supporting the “happy superiority effect” [17]. There is no consensus on which emotional crowd people are more sensitive to. In addition, the extraction of crowd emotion may be less affected and may even be independent of the visual physical features of facial elements such as brightness and contrast [18]. Notably, the spatial frequency, as an elementary source encoding visual stimuli, has been ignored in the research of ensemble processing of crowd emotion.

Spatial frequency is defined as the luminance variation over a distance unit. The characteristics of objects are visually transmitted through information in different frequency bands: low spatial frequency (LSF) mainly represents rough visual information and can be recognized quickly, whereas high spatial frequency (HSF) provides local and detailed features of the stimuli [19,20]. In line with this, previous studies have provided empirical evidence of the effect of spatial frequency on the processing of a single facial stimulus [21,22,23]. The processing of fearful and angry faces relies primarily on LSF information [24,25,26], whereas HSF plays a prominent role in the recognition of sad faces [27]. Recently, researchers found that LSF information also seems important for the recognition of happy faces [28]. However, the link between spatial frequency and the perception of crowd faces has not been directly explored thus far, being investigated in the present study.

Two visual pathways extend from the ganglion cells of the retina to the input layer of the primate visual cortex through the lateral geniculate body, namely the magnocellular pathway (the M pathway) and the parvocellular pathway (the P pathway), which mainly project to the dorsal and ventral stream, respectively [29,30,31]. The M and P pathways prefer different frequency bands of visual information; the former is more sensitive to LSF information, and the latter is better at processing HSF information [32,33,34]. Studies targeting facial stimuli have yielded consistent findings. The LSF component of fearful faces preferred to activate subcortical areas such as the amygdala, superior colliculus, and thalamus through the M pathway, whereas the HSF information contained in fearful faces preferred engagement of the fusiform gyrus through the P pathway [25,26,35]. 

In addition to spatial frequency, the two pathways manifested differential preferences for the processing of single and crowd facial stimuli. As shown by Im [36,37], brain regions located in the dorsal visual stream, primarily receiving input from the M pathway, were preferentially activated by crowd faces, especially in the intraparietal sulcus and superior frontal gyrus, whereas activity in the fusiform cortex in the ventral stream, primarily receiving input from the P pathway, predicted a better perception of individual facial expressions. Combined with the fact that the M pathway delivering rough visual information prefers LSF components [29,38,39], this indicates that LSF information critically contributes to the perception of crowd faces. Thus, we speculate that the frequency information of crowd faces can affect the perception of emotional content and that this effect may occur in some brain regions located in the dorsal visual stream.

Taken together, the current study utilized Haberman’s classical experimental design [8,40,41] in which participants compared the valence of a test face with crowd faces displaying varying emotional expressions (fearful, neutral, and happy) and spatial frequencies (LSF, BSF, and HSF; low, broad and high spatial frequency, respectively). We conducted a functional magnetic resonance imaging (fMRI) experiment to investigate how spatial frequencies impact crowd face processing and made the following predictions: (1) in terms of behavior, there is a heightened sensitivity to low spatial frequency (LSF) crowd faces, particularly when they exhibit fear, compared to high spatial frequency (HSF) crowd faces; and (2) the frequency content of crowd faces can significantly influence how they are perceived. Specifically, LSF crowd faces engage specific brain regions within the dorsal visual pathway, whereas HSF crowd faces activate distinct regions along the ventral visual pathway. This study explored the impact of essential visual elements (spatial frequency) on crowd faces recognition, providing new evidence and support for understanding the behavioral and neural patterns involved in processing crowds. Furthermore, it helps individuals identify the affective states of crowds, apply suitable strategies to integrate into the group, and even avoid threats.

## 2. Materials and Methods

### 2.1. Participants

Thirty-five undergraduate students from Liaoning Normal University participated in an experiment, but two participants were excluded from further fMRI analyses due to movement artifacts during the scanning session. The final sample comprised 33 participants, consisting of 13 males and 20 females (mean age = 20.7 years, sd. = 1.89 years) who were right-handed. All participants had normal or corrected-to-normal visual acuity and normal color vision, and there were no known cognitive or neurological impairments. Additionally, all participants provided informed written consent and received monetary compensation for their participation. The protocols were approved by the ethics committee of Liaoning Normal University.

### 2.2. Stimuli

**Figure 1:**
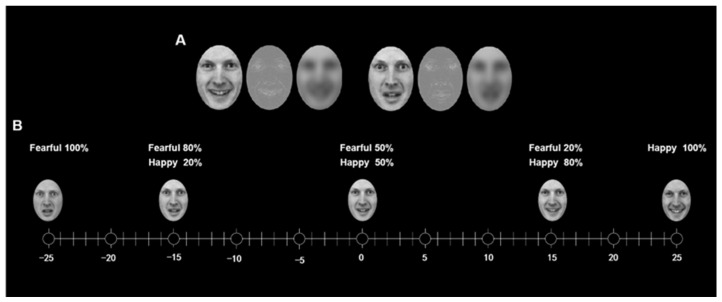
Experimental stimuli. (**A**) Happy and fearful faces with a normal broad spatial frequency (BSF) were filtered to contain only a high or low spatial frequencies (Happy: **left**; Fearful: **right**). (**B**) Face stimuli morphed from two extreme fearful and happy faces of the same person with −25 emotional units being extremely fearful, 0 being neutral, and +25 being extremely happy.

Stimuli we made were adapted from the previous studies on the ensemble coding of faces and the recognition of crowd emotion [8,37]. The happy and fearful facial expressions of the male numbered AM10 from the Karolinska Directed Emotional Faces (KDEF) were selected as the original experimental material [42]. We created a set of 51 faces using face-morphing software (Abrosoft FantaMorph 5.4.5) by linearly interpolating (in 2% increments) between two highly intense prototypical facial expressions. The resulting faces were separated by emotional units of intensity such that face 2 was one emotional unit happier than face 1 (see Figure 1). In the face set, 0 meant neutral (e.g., a morph of 50% happy and 50% fearful), +9 meant happier (e.g., a morph of 68% happy and 32% fearful) and −9 meant more fearful (e.g., a morph of 32% happy and 68% angry). Since the previous literature on the averaging of faces showed that the range of variation was important, we kept the range of faces about 18 units across the crowds [37]. To generate the LSF and HSF face images, we first transformed the morphed faces to grayscale, normalized them to equal luminance, and then applied either low-pass filtering at 2 cycles per degree or high-pass filtering at 6 cycles per degree, respectively. The low-and high-pass cut-offs were determined with reference to prior research [43,44,45,46] (Figure 1). Filtering was conducted in MatlabR 2018b (Mathworks, Natick, MA, USA), using a set of two-order Butterworth filters. Subsequently, we used the SHINE toolbox to standardize the luminance and contrast of all the filtered image sets [47]. 

Then, the single-face expressions, which have been morphed and filtered as described above, were formed crowd faces. The crowd faces consisted of four single faces with the same spatial frequency of happiness or fear, and the mean emotion was selected at −9, −3, 0, 3, or 9 emotional units, with positive values indicating a happier crowd emotion compared to the neutral and negative values indicating a more fearful mean emotion. Only one main emotion was used per trial (e.g., happy). Once the mean emotion was selected, four unique facial expressions were chosen surrounding the mean, which were each separated by at least 6 emotional units [8,18]. For instance, if high spatial frequency crowd faces with a mean emotion of +9 were selected, the four faces comprising the crowd corresponded to the emotional units of high spatial frequency 0, +6, +12, and +18. Each face image subtended 3.04° × 4.34° of the visual angle, and the face sets consisted of four items presented on the screen in a grid pattern measuring 6.94° × 9.53°.

### 2.3. Procedure

In this experiment, we used the classical task of ensemble coding in a crowd of faces [8,48,49]. At the beginning of each trial, the white cross was presented for 500 ms, and then the crowd faces were displayed for 2000 ms, which were followed by the white cross for 500 ms. Finally, a single test face was presented, which had an emotion 9 units higher or lower than the mean emotion of the preceding crowd. In the experiment, there were 9 types of crowd faces, including higher spatial frequency (HSF), broad spatial frequency (BSF), and lower spatial frequency (LSF) under happy, neutral, and fearful conditions. All test faces were either 9 units below or 9 units above the mean emotion of the crowd faces. Haberman and Whitney [18] demonstrated that this manipulation allowed for the differentiation of emotion between the test face and the mean emotion of the crowd faces. 

**Figure 2:**
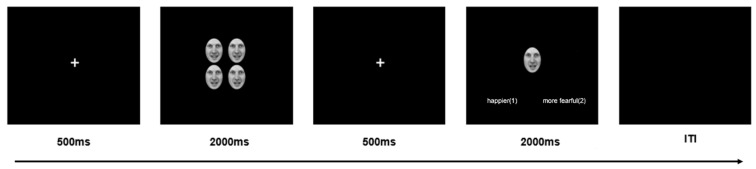
Sample trial of experiment. The stimuli presented were BSF happy crowd faces.

Participants were instructed to distinguish whether the test face conveyed a happier or more fearful expression compared to the crowd by pressing the ‘1′ key for happier and the ‘2′ key for more fearful, using a button box. The response time was limited to 2000 ms. The intertrial interval (ITI) ranged randomly between 1000 and 7000 ms (see Figure 2). During stimulus presentation periods, participants were required to maintain visual fixation at the center of the screen. To ensure that participants clearly understood the procedure, a practice stage consisting of 36 trials was conducted. The main experiment was divided into 5 blocks, with short rest periods between blocks. Each block contained 72 trials with an equal number of trials for each condition presented in random order, resulting in a total of 360 trials. The experiment was run using E-Prime 2.0.10.182 software (Psychology Software Tools Inc., Pittsburgh, PA, USA).

### 2.4. Behavioral Data Analysis

Behavioral data were statistically analyzed using jamovi 1.2.27.0 [50] (https://www.jamovi.org). Response times exceeding 3 standard deviations from the mean were considered late and excluded from the data analyses. Because the participants reported the emotion judgement task was somewhat difficult, a one-sample *t*-test (vs. 50% chance level) was performed on the accuracy of all participants, and then the two-way repeated measures analysis of variance (ANOVA) was conducted to examine the effects of emotion (happy, neutral and fear) and spatial frequency (HSF, BSF and LSF) for accuracy (ACC) and response time (RT). If the test face was 9 units higher than the mean emotion of crowd faces, it was judged to be happier. Conversely, the lower 9 units were correct for judging more fear. The significance level was set at *p*= 0.05 for all the analyses. The Greenhouse–Geisser correction was conducted to account for sphericity violations whenever appropriate. The Bonferroni correction was applied to corrected false positive errors caused by multiple comparisons of post hoc testing of the significant effects. Additionally, we calculated sensitivity (true positives/(true positives plus false negatives)) and specificity (true negatives/(true negatives plus false positives)) to supplement the behavioral analysis; see the Supplementary Materials.

### 2.5. fMRI Data Acquisition and Analysis

The Brain Imaging Center at Liaoning Normal University conducted MRI imaging using a 3-Tesla MRI scanner (Discovery MR750 3.0T, GE Healthcare, Chicago, IL, USA) and an 8-channel head coil. During the experiment, subjects were positioned flat on the machine, fixed their heads, remained still throughout the procedure, and used earplugs to minimize scanning noise interference. To minimize head movement, a foam pad compatible with the MRI machine was used to fix each subject’s head. The T1-weighted images were acquired sagittal using the following parameters: repetition time (TR) = 1900 ms; echo time (TE) = 2.52 ms; flip angle = 9°; the field of view (FOV) = 256 mm × 256 mm; matrix size = 256 × 256; voxel size = 1 × 1 × 1 mm^3^; slice thickness = 1 mm and 176 slices. Then, the T2-weighted images were acquired utilizing the echo-planar imaging (EPI) BOLD sequence with the following imaging settings: repetition time (TR) = 2000 ms; echo time (TE) = 30 ms; flip angle = 90°; the field of view (FOV) = 192 mm × 192 mm; matrix size = 64 × 64; voxel size = 3 × 3 × 3 mm^3^; slice thickness = 3 mm and 43 slices.

The data were pre-processed and analyzed using SPM 12 (Statistical Parametric Mapping, Wellcome Department of Cognitive Neurology) [51] (http://www.fil.ion.ucl.ac.uk/spm/). The pre-processing steps included the removal of the first 3 volumes, slice-timing correction, head motion estimation and correction (>2 mm in any plane were discarded), non-linear normalization to the standardized Montreal Neurological Institute (MNI) space, and spatial smoothing with a Gaussian kernel filter of 6 mm full-width half-maximum kernel. 

We performed a first-level analysis on individual data using the General Linear Model (GLM) for an event-related design. The analysis included 9 experimental event types: HSF—neutral crowds, BSF—neutral crowds, LSF—neutral crowds, HSF—fearful crowds, BSF—fearful crowds, LSF—fearful crowds, HSF—happy crowds, BSF—happy crowds, and LSF—happy crowds. We also included each participant’s wrong response time, the presentation time of the test stimulus, and head movement-related variance and realignment parameters (x, y, and z translations and pitch, roll, and yaw rotations) in the model. These variables were modeled and convolved with the canonical HRF function. Each experimental condition was modeled with its onset starting after the stimulus appearance until the end of the stimulus presentation. We then estimated the model and calculated contrast images for each experimental condition. These contrast images were entered into group-level statistics. 

For group analysis, these estimates were then entered into a second-level analysis with the two-way repeated ANOVA at each voxel using the 3 (emotion: happy crowds, neutral crowds, fearful crowds) × 3 (spatial frequency: HSF crowds, BSF crowds, LSF crowds) experimental design. We applied whole-brain family-wise error correction (FWE) to all the reported data, using a significance threshold of *p* < 0.05, and a minimum cluster size of 10 voxels.

## 3. Results

### 3.1. Behavioral Results

Despite some reported difficulty in making judgments, the participants performed above chance level. Overall, the experiment’s accuracies were significantly higher than chance [*t* (32) = 19.4, *p* < 0.001], indicating that the participants were able to extract the average emotion from the set. 

Accuracy: Results showed significant effects of emotion [*F* (2, 64) = 30.71, *p* < 0.001, *η_p_*^2^ = 0.49] and spatial frequency [*F* (2, 64) = 41.18, *p* < 0.001, *η_p_*^2^ = 0.56] on accuracy. Post hoc comparisons revealed that neutral crowds had higher accuracy (M ± SE, 0.824 ± 0.017) than fearful crowds [0.788 ± 0.017, *t* (64) = 3.09, *p* < 0.01] and happy crowds [0.723 ± 0.014, *t* (64) = 7.78, *p* < 0.001]. Fearful crowds had significantly higher accuracy than happy crowds [*t* (64) = 4.69, *p* < 0.001]. Regarding spatial frequency, accuracy was higher for BSF (0.867 ± 0.013) than for LSF [0.779 ± 0.018, *t* (64) = 9.07, *p* < 0.001] and HSF [0.698 ± 0.022, *t* (64) = 4.71, *p* < 0.001]. Additionally, LSF accuracy was significantly higher than HSF accuracy [*t* (64) = −4.37, *p* < 0.001]. Moreover, the results of sensitivity and specificity were almost consistent with ACC, which are presented in the Supplementary Materials.

**Figure 3:**
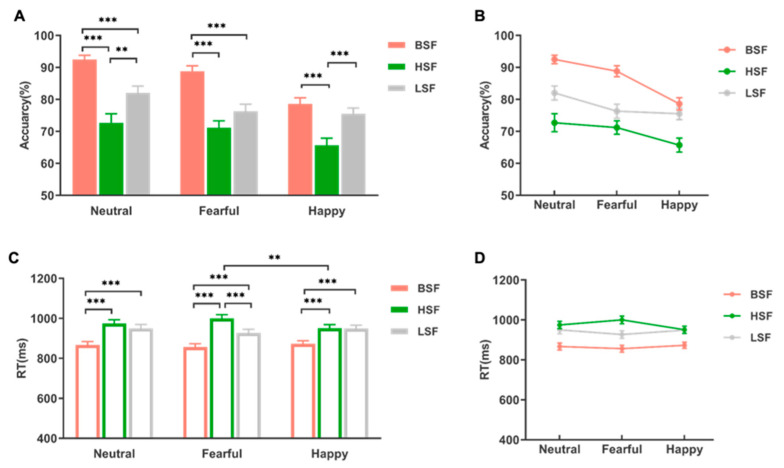
Behavioral results of the experiment. (**A**,**B**) The effect of emotion × spatial frequency for accuracy. (**C**,**D**) The effect of emotion × spatial frequency for RT. Error bars represents standard errors. (** *p* < 0.01, *** *p* < 0.001).

The interaction of emotion × spatial frequency was also significant [*F* (4, 128) = 6.13, *p* < 0.001, *η_p_*^2^ = 0.16]. Simple effect analyses indicated that in the BSF condition, neutral crowds had higher accuracy (0.925 ± 0.013) than in the LSF condition [0.820 ± 0.022, *t* (123) = 4.70, *p* < 0.001] and HSF condition [0.727 ± 0.028, *t* (123) = 8.89, *p* < 0.001]. The LSF condition was also significantly higher than the HSF condition (*p* < 0.05). For happy crowds, the BSF condition resulted in higher accuracy (0.786 ± 0.019) than the HSF condition [0.657 ± 0.022, *t* (123) = 5.80, *p* < 0.001], and the LSF condition was significantly higher than the HSF condition [0.755 ± 0.018, *t* (123) = −4.38, *p* < 0.001]. Fearful crowds had higher accuracy in the BSF condition (0.931 ± 0.013) than in the LSF condition [0.789 ± 0.020, *t* (123) = 5.58, *p* < 0.001] and the HSF condition [0.908 ± 0.014, *t* (123) = 7.89, *p* < 0.001], but there was no significant difference between the LSF and HSF conditions [*t* (123) = 4.70, *p* = 0.825] (see Figure 3).

Response Time (RT): We removed the errors and the trials with more than three standard deviations of response time and then analyzed the remaining. The results showed a significant main effect of spatial frequency [*F* (2,64) = 70.26, *p* < 0.001, *η_p_*^2^ = 0.69] and a significant interaction between emotion and spatial frequency [*F* (4,128) = 6.22, *p* < 0.001, *η_p_*^2^ = 0.16], but no significant main effect of emotion [*F* (2,64) = 0.47, *p* = 0.627, *η_p_*^2^ = 0.001]. Post hoc pairwise comparisons indicated that the response time differed significantly among the three conditions [BSF vs. HSF: *t* (64) = −11.56, *p* < 0.001; BSF vs. LSF: *t* (64) = −8.05, *p* < 0.001; HSF vs. LSF: *t* (64) = 3.50, *p* < 0.05]. Response time was shortest for crowd faces with BSF (865 ± 15.7 ms), which was followed by LSF (942 ± 16.3 ms), and it was longest for HSF (975 ± 17.1 ms). Further analysis of the emotion × spatial frequency interaction effect revealed that the response time for neutral and happy crowds in the BSF condition (neutral: 867 ± 17.1 ms; happy: 873 ± 15.5 ms) was significantly faster than in the LSF [neutral: 950 ± 19.2 ms, *t* (170) = −6.21, *p* < 0.001; happy: 949 ± 17.1 ms, *t* (170) = −5.67, *p* < 0.001] and HSF [neutral: 975 ± 18.1 ms, *t*(170) = −8.06, *p* <0.001; happy: 951 ± 17.8 ms, *t* (170) = −5.80, *p* < 0.001] conditions. However, response time did not significantly differ between the LSF and HSF conditions for neutral and happy crowds (*p* = 1.00). The speed at which fearful crowds were identified was significantly faster in the BSF condition (856 ± 16.9 ms) compared to both the LSF condition [927 ± 18.7 ms, *t* (170) = −5.294, *p* < 0.001] and the HSF condition [1000 ± 18.7 ms, *t* (170) = −10.77, *p* < 0.001]. Additionally, there was a significant difference in identification speed between the LSF and HSF conditions [*t* (170) = 5.48, *p* < 0.001]. It was worth noting that in the HSF condition, happy crowds were identified significantly slower than fearful crowds [*t* (192) = 4.23, *p* < 0.001] (see Figure 3). The specific behavioral results are summarized in Table 1.

### 3.2. fMRI Results

**Figure 4:**
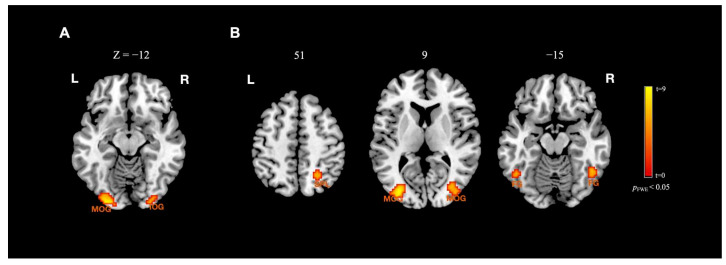
Brain activations by HSF crowd faces comparisons. (**A**) Brain areas that showed greater activations to HSF crowd faces as compared to LSF crowd faces. (**B**) Brain areas that showed greater activations to HSF crowd faces as compared to BSF crowd faces. Clusters threshold: *p* < 0.05 (cluster-level FWE correction). Abbreviations: MOG, middle occipital gyrus; IOG, inferior occipital gyrus; SPL, superior parietal lobule; FG, fusiform gyrus.

**Figure 5:**
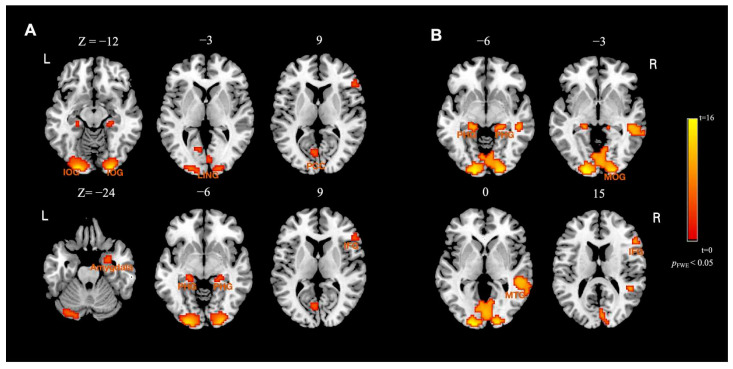
Brain activations by BSF crowd faces comparisons. (**A**) Brain areas that showed greater activations to BSF crowd faces as compared to LSF crowd faces. (**B**) Brain areas that showed greater activations to BSF crowd faces as compared to HSF crowd faces. Clusters threshold: *p* < 0.05 (cluster-level FWE correction). Abbreviations: IOG, inferior occipital gyrus; LING, lingual gyrus; PHG, parahippocampal gyrus; PCC, posterior cingulate gyrus; MOG, middle occipital gyrus; IFG, inferior frontal gyrus; MTG, middle temporal gyrus.

In our fMRI experiment, we aimed to identify the neural substrates involved in processing crowd faces with different spatial frequency information. Our whole-brain analysis revealed that HSF crowds elicited greater cortical responses compared to LSF crowds in the left middle occipital gyrus (MOG) (x = −24, y = −93, z = −12, *t* = 9.05) and right inferior occipital gyrus (IOG) (x = 27, y = −93, z = −12, *t* = 6.32). Additionally, when comparing HSF and BSF crowds, we observed greater activations in bilateral MOG (left: x = −33, y = −81, z = 9, *t* = 8.62; right: x = 39, y = −84, z = 12, *t* = 7.20), bilateral fusiform gyrus (left: x = −48, y = −57, z = −15, *t* = 5.65; right: x = 48, y = −54, z = −15, *t* = 6.23), and right superior parietal lobule (SPL) (x = 24, y = −57, z = 51, *t* = 5.87) (see Figure 4). Moreover, the contrast of BSF crowds minus LSF crowds revealed greater activations in bilateral IOG (left: x = −24, y = −93, z = −12, t = 16.90; right: x = 24, y = −93, z = −12, *t* = 16.58) as well as in the left posterior cingulate gyrus (x = −6, y = −69, z = 9, *t* = 5.79), right lingual gyrus (x = 3, y = −81, z = −3, *t* = 5.20), bilateral parahippocampal gyrus (left: x = −21, y = −30, z = −6, *t* = 6.41; right: x = 24, y = −30, z = −9, *t* = 6.05), right amygdala (x = 24, y = 0, z = −24, *t* = 5.83), and right inferior frontal gyrus (IFG) (x = 54, y = 33, z = 9, t = 5.69). Lastly, when comparing BSF and HSF crowds, we found more activations in the bilateral parahippocampal gyrus (left: x = −18, y = −33, z = −6, *t* = 6.22; right: x = 21, y = −30, z = −9, *t* = 5.84), right middle temporal gyrus (MTG) (x = 57, y = −36, z = 0, *t* = 6.59), right IFG (x = 54, y = 27, z = 15, *t* = 5.71), and right regions of the occipital lobe (x = 18, y = −96, z = −3, *t* = 9.48) (e.g., lingual gyrus, MOG, IOG, fusiform gyrus, etc.) (see Figure 5). The complete list of activations is reported in Table 2.

## 4. Discussion

Previous studies have demonstrated the influence of spatial frequency on the perception of individual facial expressions. Based on neurobehavioral findings regarding crowd faces, the current study extended the effect of spatial frequency to the processing of crowd emotion. First, our findings revealed that fearful crowds were easier to recognize than happy crowds. Notably, neutral crowd faces were the best recognized in the current study (Acc: 82.4%), which may have resulted from the fMRI design in which the neutral crowd faces were repeated twice, guaranteeing a balance between the experimental conditions. Moreover, compared with HSF information, LSF information was more helpful for crowd emotion perception, especially of fearful faces. Second, brain regions located in the ventral visual stream (e.g., fusiform gyrus) were preferentially activated in HSF crowds; however, their behavior was the most difficult to recognize. HSF does not promote crowd face recognition.

### 4.1. Impact of Spatial Frequency on Behaviors

Compared to HSF faces (Acc:69.8%, RT: 975 ms), the perception of LSF faces was easier (Acc: 77.9%), quicker (RT: 942 ms), and sensitive. The broad spatial frequency (BSF) faces had the highest recognition rate (Acc:86.7%) and fastest reaction speed (RT: 865 ms). In addition, sensitivity and specificity were also significantly higher than those of HSF because they contained broad spatial frequency information. The LSF information transmits the rough contoured content of the face and is more conducive to the judgment of expression, as demonstrated in previous studies on the stimuli of a single face [45,46,52], which was extended to crowd faces in the current study. However, HSF crowd faces included local and detailed information and were more difficult and slower to recognize behaviorally. In line with the dual-route model of emotion processing, there are two parallel routes for the processing of emotional information: a subcortical “low road” that provides fast, but crude, biologically significant signals to the amygdala, and a longer, slower “high road” that processes detailed information through cortical visual areas [53,54]. 

Notably, the contribution of spatial frequency, as indicated by our results, seems to be dependent on the type of emotion. While the effect of LSF versus HSF on emotional recognition accuracy disappeared for fearful crowd faces, it displayed differences in terms of reaction time. This may be a result of the priority of threat information delivered by fearful faces [14,55,56], which promotes the reaction speed to fear due to its coherence with LSF information, being supported by the lower specificity. Our findings align with neural computational research demonstrating that LSF information is more effective than HSF content in facilitating the categorization of threat-relevant faces [57,58]. The rapid and sensitive capture of fearful information, however, introduces more noise, which, to some extent, limits the increase in accuracy and specificity in recognizing emotions within crowd faces.

Participants showed a better and more sensitive performance in the perception of fearful crowd faces, which is consistent with the findings regarding single faces [14,55,59]. Fenker et al. [12] reported that people were easily distracted by fearful faces. Luo et al. [14] also suggested that accuracy and early event-related potential (ERP) components were lager with fearful rather than with happy and neutral faces in deficient attention. First, fear can prevent individuals from becoming conspicuous targets of aggressive species, making them seem less dominant and therefore less likely to be harmed. Second, fear sends danger signals and protects others [59]. Remarkably, as socially connected species, humans are more sensitive and focused to information delivered by crowds, which can guide and even change behavior unconsciously [60,61]. The “crowd emotion amplification effect”, in which a crowd’s average emotional response is perceived as more extreme than it is [62,63], may contribute to the perceptional bias of fearful crowd faces. Fear information that is closely related to people’s survival and development is more easily perceived and recognized by individuals after being amplified by the crowd. To summarize, this finding enriched the content of facial emotion perception from the perspective of “ensemble coding and crowd emotion.”

### 4.2. Impact of Spatial Frequency on Neural Patterns

The spatial frequency comparison evoked greater activation in various brain areas. HSF crowds significantly activated the bilateral MOG and IOG (versus LSF crowds) and the bilateral MOG, MTG, fusiform gyrus, and SPL (versus BSF), which is partly consistent with the findings of previous studies revealing the effect of HSF in the bilateral IOG, left inferior temporal gyrus (ITG), right fusiform gyrus [26,64], and left occipitotemporal cortex [65]. These brain areas are mostly located in the ventral visual stream, receive input from the P pathway, and neurally represent the “what” information of crowd faces [66,67]. Although HSF within crowds may represent more concrete and specific information, it was the most difficult to recognize behaviorally in our study. This information cannot significantly contribute to the “ensemble coding” underlying the perception of crowd faces, which mostly depends on the rapid average of the visual features [9,18]. The BSF crowds contained abundant spatial frequency content and elicited stronger activation in the right occipital lobe, bilateral parahippocampal gyrus, and right IFG than the HSF and LSF crowds. The parahippocampal cortex is highly engaged in tasks involving spatial information (e.g., spatial frequency), such as viewing pictures of landscapes and surrounding buildings using spatial maps and object locations [68,69,70]. Our results verify the sensitivity of the parahippocampal gyrus to spatial information, particularly in response to visual facial stimuli. The anterior lingual gyrus is attached to the parahippocampal gyrus, which is engaged in the primary processing of visual information and facial expression recognition [71]. Additionally, we observed greater activation evoked by BSF crowds in the right IFG, which is responsible for inhibitory control and attentional demands [72,73]. This may suggest a greater demand of other cognitive centers for BSF crowds than for crowds with partial frequency information.

Im [37] showed that the right hemisphere had greater participation in the processing of crowd faces when the task goal matched the emotional valence or social motivation of the stimulus. Similarly, we observed that the brain regions of the right hemisphere seemed to be more pronounced in processing facial crowds regardless of the spatial frequency conditions, suggesting that the right hemisphere may be more engaged in crowd face perception. Despite the observation of a bias in recognizing fearful crowd faces and greater sensitivity to LSF information in behavioral data, these phenomena were not reflected in brain activity patterns. Our analysis focused on the brain activity of participants while passively viewing faces, followed by subsequent emotion judgment, which may have contributed to the lack of discernible emotional differences in brain activity. This does not necessarily indicate the absence of emotional processing of crowd faces in the brain. Rather, the results suggest that the brain tends to be attracted to the physical characteristics of crowd faces and displays less sensitivity to emotional information when faced with crowd stimuli, differing from individual faces whose emotional information preferentially captures attention [55,74].

### 4.3. Possible Moderators and Limitations

Attention may be an important factor to modulate the impact of spatial frequency on the perception of crowd emotion. Tian, Wang, Xia, Zhao, Xu and He [46] reported that LSF emotional (happy and fearful) faces were distinguished from neutral faces at an early stage with limited attention. Furthermore, subcortical regions, including the amygdala, superior colliculus, and thalamus, exhibited heightened activation in response to the LSF fearful face [26]. These findings imply a rapid and effective transmission of coarse-grained emotion information, even at an unconscious level. 

However, it is yet unknown whether attention modulates the impact of spatial frequency on crowd emotion. Some researchers supported that the extraction of mean emotion from crowd faces was fast and automatic [75,76]. Even if faces are presented for 50 ms, the accuracy of the recognition of crowd emotion was significantly higher than chance [76]. When attention is diffused or insufficient, individual items cannot be accurately represented; however, multiple items can be averaged and integrated into a relatively precise ensemble representation [9]. The ensemble coding not only applies to emotion perception but also other crowd face characteristics, such as identity [8,18] and attractiveness [77]. By contrast, Mcnair et al. [78] found a significant attentional blink effect in the recognition of crowd emotions. The accuracy under a short lag was significantly lower than that under a long lag, implying that attention resources affect the extraction of emotions. However, which spatial frequency information is crucial for crowd emotion recognition when attention is limited? Does LSF information facilitate the processing of crowd emotion, especially in fearful crowds? These issues deserve further thought and exploration. 

This study has several limitations. First, attention may modulate the impact of spatial frequency on crowd emotion, just as discussed. However, we did not control this factor in our experiments, and it may be further investigated by adjusting the presentation time, increasing the number of faces, and manipulating experimental paradigms. Second, considering more experimental factors may confuse the results, we avoided a larger array of emotions, focusing only on fear (representing negative emotion) and happiness (representing positive emotion), adding neutral crowds as a baseline. In the real world, facial expressions convey complex and rich social signals. Even if the valence is all negative, the motivation and social intention expressed are different. For example, anger often means avoidance [79]; however, fear and sadness imply approach [80,81]. Future research can increase the range of emotion types (e.g., angry or sad) to prove the generality of other emotional expressions. Third, previous research has indicated that men tend to be more sensitive to angry faces, whereas women exhibit a greater inclination to process happy faces [82]. In this study, we only used male stimulus. Further studies should consider the use of a more diverse set of facial identities, improving the ecological validity of the results. Finally, subsequent research can draw insights from playing videos [83] or other more ecologically valid methods, such as virtual reality (VR), to enhance the generalizability of the experimental outcomes. 

## 5. Conclusions

The current findings provide evidence that spatial frequency affects crowd emotion processing: (1) the behavioral methods prove that fearful faces with low spatial frequency are easier to recognize, which expands existing research on individual faces; (2) high spatial frequency crowds do not seem to promote crowd face recognition from the perspective of brain representation; and (3) faces with normal spatial frequency in neural patterns need to use more attention resources. Overall, our study suggests that people are more sensitive to fearful crowd faces with low spatial frequency and that high spatial frequency does not promote crowd face recognition.

## Figures and Tables

**Table 1:** Statistical results of behavioral data from a two-way repeated-measures ANOVA. Notes: Effects significant at an alpha level of 0.05 are shown in bold font.

	Effect	ANOVA Results			
		*F*	*p*	*η_p_* ^2^	Post Hoc Tests
ACC	Emotion	**30.71**	**<0.001**	**0.49**	Neutral > Fearful > Happy
	Spatial frequency	**41.18**	**<0.001**	**0.56**	BSF > LSF > HSF
	Emotion × Spatial frequency	**6.13**	**<0.001**	**0.16**	Neutral: BSF > LSF > HSF
					Happy: BSF > LSF > HSF
					Fearful: BSF > LSF/HSF
RT	Emotion	0.47	0.627	0.001	
	Spatial frequency	**70.26**	**<0.001**	**0.69**	BSF < LSF < HSF
	Emotion × Spatial frequency	**6.22**	**<0.001**	**0.16**	Neutral: BSF < LSF/HSF
					Happy: BSF < LSF/HSF
					Fearful: BSF < LSF < HSF

**Table 2:** Significantly activated areas in mean responses for different spatial frequency contrasts. Clusters threshold: *p* < 0.05 (cluster-level FWE correction).

Activation Location	MNI Coordinates			*t*	Cluster Size
	x	y	z		
***HSF > LSF***					
Left middle occipital gyrus	−24	−93	−12	9.05	117
Right inferior occipital gyrus	27	−93	−12	6.32	31
***HSF > BSF***					
Left middle occipital gyrus	−33	−81	9	8.62	94
Right middle occipital gyrus	39	−84	12	7.20	65
Right superior parietal lobule	24	−57	−15	5.87	23
Right fusiform gyrus	48	−54	−15	6.23	19
Left fusiform gyrus	−48	−57	−15	5.65	12
***BSF > LSF***					
Left inferior occipital gyrus	−24	−93	−12	16.90	297
Right inferior occipital gyrus	24	−93	−12	16.58	189
Left posterior cingulate gyrus	−6	−69	9	5.79	22
Right lingual gyrus	3	−81	−3	5.20	20
Left parahippocampal gyrus	−21	−30	−6	6.41	18
Right parahippocampal gyrus	24	−30	−9	6.05	17
Right amygdala	24	0	−24	5.83	17
Right inferior frontal gyrus	54	33	9	5.69	11
***BSF > HSF***					
Right occipital lobe	18	−96	−3	9.48	710
Right lingual gyrus	24	−93	−9	9.41	168
right middle temporal gyrus	57	−36	0	6.59	133
Left parahippocampal gyrus	−18	−33	−6	6.22	23
Right parahippocampal gyrus	21	−30	−9	5.84	22
Right inferior frontal gyrus	54	27	15	5.71	16

## Data Availability

The data are available from the corresponding author upon reasonable request.

## Supplementary Materials

The following supporting information can be downloaded at: https://www.mdpi.com/article/10.3390/brainsci13121699/s1, Figure S1: Sensitivity and specificity results of the experiment.; Table S1: Statistical results of sensitivity and specificity from a two-way repeated measures ANOVA.
