# Supplementary material for: The Impact of Spatial Frequency on the Perception of Crowd Emotion: An fMRI Study
Source: Brain Sci. 2023 Dec 9;13(12):1699. doi: 10.3390/brainsci13121699 (PMC10742193; doi:10.3390/brainsci13121699)
Supplement: Supplementary file 1 [file brainsci-13-01699-s001.zip › brainsci-2679795-supplementary.pdf]

Sensitivity and Specificity analysis and results

1. *Sensitivity and Specificity analysis*

We computed sensitivity (true positives/ [true positives plus false negatives]) and specificity (true negatives/ [true negatives plus false positives]) for each condition. The true positives included the number of more fearful face correctly recognized, whereas the true negatives consisted of the number of happier faces correctly classified as such. Then, the two-way repeated measures analysis of variance (ANOVA) was conducted to examine the effects of emotion (happy, neutral and fear) and spatial frequency (HSF, BSF and LSF) for sensitivity and specificity. The significance level was set at P=0.05 for all the analyses. The Greenhouse–Geisser correction was conducted to account for sphericity violations whenever appropriate. The Bonferroni correction was applied to corrected false positive errors caused by multiple comparisons of post hoc testing of the significant effects.

2. *Sensitivity and Specificity results*

Sensitivity: Results showed significant effects of emotion [F (2, 64) = 104.93, $p < 0.001$, $\eta_p^2 = 0.77$] and spatial frequency [F (2, 64) = 29.91, $p < 0.001$, $\eta_p^2 = 0.48$]. The interaction of emotion × spatial frequency was also significant [F (4, 128) = 3.39, $p < 0.05$, $\eta_p^2 = 0.10$]. The post hoc comparisons and specific results were summarized in Supplementary Table S1 and Supplementary Figure S1.

Specificity: The effects of emotion [F (2, 64) = 98.72, $p < 0.001$, $\eta_p^2 = 0.76$] and spatial frequency [F (2, 64) = 14.19, $p < 0.001$, $\eta_p^2 = 0.31$] were significant. The interaction of emotion × spatial frequency was also significant [F (4, 128) = 5.24, $p < 0.001$, $\eta_p^2 = 0.14$]. The post hoc comparisons and specific results were summarized in Supplementary Table S1 and Supplementary Figure S1.

	Effect	ANOVA results			
		**F**	**p**	**η_p^2**	**Post hoc tests**
Sensitivity	Emotion	**104.93**	**< 0.001**	**0.77**	Fearful >Neutral >Happy
	Spatial frequency	**29.91**	**< 0.001**	**0.48**	BSF /LSF > HSF
	Emotion × Spatial frequency	**3.39**	**< 0.05**	**0.10**	Neutral: BSF /LSF > HSF
					Happy: LSF > HSF
					Fearful: BSF /LSF>HSF
Specificity	Emotion	**98.72**	**< 0.001**	**0.76**	Happy>Neutral> Fearful
	Spatial frequency	**14.19**	**< 0.001**	**0.31**	BSF > LSF /HSF
	Emotion × Spatial frequency	**5.24**	**< 0.001**	**0.14**	Neutral: BSF >LSF/HSF
					Happy: BSF > LSF/HSF
					Fearful: BSF > HSF > LSF

Supplementary Table S1. Statistical results of sensitivity and specificity from a two-way repeated measures ANOVA. Notes. Effects significant at an alpha level of 0.05 are shown in bold font.

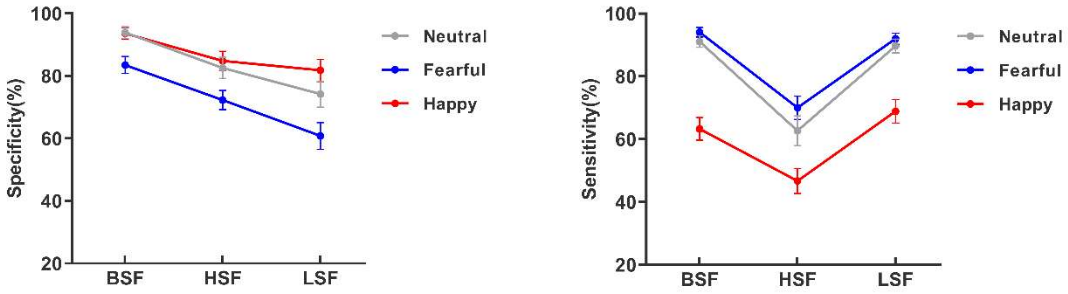

Supplementary Figure S1. Sensitivity and specificity results of the experiment.
